# Clinical Manifestations, Current and Future Therapy, and Long-Term Outcomes in Congenital Thrombotic Thrombocytopenic Purpura

**DOI:** 10.3390/jcm12103365

**Published:** 2023-05-09

**Authors:** Kazuya Sakai, Masanori Matsumoto

**Affiliations:** 1 Department of Blood Transfusion Medicine, Nara Medical University, Kashihara 634-8522, Japan; ks13122@naramed-u.ac.jp; 2 Department of Hematology, Nara Medical University, Kashihara 634-8521, Japan

**Keywords:** congenital thrombotic thrombocytopenic purpura, ADAMTS13, fresh frozen plasma, prophylaxis, quality of life

## Abstract

Congenital thrombotic thrombocytopenic purpura (cTTP) is an extremely rare disease characterized by the severe deficiency of a disintegrin and metalloproteinase with thrombospondin type 1 motifs 13 (ADAMTS13), caused by *ADAMTS13* mutations. While ADAMTS13 supplementation by fresh frozen plasma (FFP) infusion immediately corrects platelet consumption and resolves thrombotic symptoms in acute episodes, FFP treatment can lead to intolerant allergic reactions and frequent hospital visits. Up to 70% of patients depend on regular FFP infusions to normalize their platelet counts and avoid systemic symptoms, including headache, fatigue, and weakness. The remaining patients do not receive regular FFP infusions, mainly because their platelet counts are maintained within the normal range or because they are symptom-free without FFP infusions. However, the target peak and trough levels of ADAMTS13 to prevent long-term comorbidity with prophylactic FFP and the necessity of treating FFP-independent patients in terms of long-term clinical outcomes are yet to be determined. Our recent study suggests that the current volumes of FFP infusions are insufficient to prevent frequent thrombotic events and long-term ischemic organ damage. This review focuses on the current management of cTTP and its associated issues, followed by the importance of upcoming recombinant ADAMTS13 therapy.

## 1. Introduction

Thrombotic thrombocytopenic purpura (TTP) is an extremely rare disease clinically characterized by severe thrombocytopenia, hemolytic anemia, and ischemic organ damage [1,2,3]. The estimated incidence of TTP is 2–6 per million persons [4]. In patients with TTP, the levels of the von Willebrand factor (VWF)-cleaving protease ADAMTS13 are severely decreased. This leads to secreted VWF remaining as an uncleaved large multimetric form called an ultra-large VWF multimer (UL-VWFM), which captures circulating platelets under high shear force via interactions between the VWF A1 domain and the GpIb domain of platelets [5,6]. As a result, VWF/platelet-rich thrombi occlude the systemic microvasculature, leading to life-threatening ischemic organ crises such as stroke and myocardial infarction [7,8]. Either autoantibodies against ADAMTS13 or causative *ADAMTS13* mutations in a homozygous or compound heterozygous state cause severe ADAMTS13 depletion in patients with TTP, which can be classified into immune-mediated TTP (iTTP) [9,10] or congenital TTP (cTTP) [11,12], also known as Upshaw–Schulman syndrome (USS), respectively. cTTP accounts for <5% of all TTP cases [13].

In 1960, Schulman et al. first described the condition of an 8-year-old girl with persistent thrombocytopenia (<100 × 10^9^/L) and repeated bleeding symptoms as “chronic thrombocytopenia”. Her platelet count responded to 6 mL/kg of fresh frozen plasma (FFP) infusions but decreased after 12 days [14]. Subsequently, Upshaw also reported an identical case of a 29-year-old woman who frequently developed high fever, petechial rash, and severe thrombocytopenia triggered by acute viral or bacterial infections. FFP infusion dramatically improved severe thrombocytopenia and normalized platelet counts. While this patient showed similar laboratory findings to typical TTP, the clinical presentation did not fulfill the five classic pentads of iTTP [15]. At that time, this disease, named after these two researchers, was not considered typical TTP, in which the disease mortality exceeded 90% without effective treatment [16,17]. Levy et al. successfully found several *ADAMTS13* mutations among USS families, leading to the recognition of USS as cTTP [11]. In the mid-2000s, causative *ADAMTS13* mutations were identified over the ADAMTS13 domains without a specific hot spot [18,19,20,21]. ADAMTS13 supply through FFP infusion is sufficient to resolve acute episodes in patients with cTTP [14,15,22], while patients with iTTP require intensive plasma exchange with FFP and immunosuppressors to survive.

Several TTP reference centers have established nationwide or international cTTP cohorts to clarify demographic characteristics, variations in causative *ADAMTS13* mutations, current ADAMTS13 replenishment, and TTP-related organ damage [18,19,20,21]. So far, more than 200 *ADAMTS13* mutations have been reported to cause cTTP. Furthermore, the anti-thrombotic effect of ADAMTS13 has been reported in experimental thrombotic mice, and the imbalanced VWF-ADAMTS13 axis has gained more attention in diverse areas of research.

The ultra-rarity of cTTP has prevented the full characterization of the clinical manifestations and optimal patient care. Since >90–95% of patients with TTP are diagnosed with iTTP, many physicians may assume that the following points are true for “all” TTP patients: (i) severe thrombocytopenia, hemolytic anemia, and ischemic organ damage occur when ADAMTS13 levels fall <10%; (ii) without suitable therapeutic interventions, >90% of cases lead to a fatal outcome; (iii) all acute and recurrent cases must be treated in the hospital, sometimes in an intensive care unit; and (iv) treatment options are expanding and there are relatively well-established treatment protocols. These four points are recognized in the field of iTTP [1,2,3]. However, although severe ADAMTS13 deficiency is present in both iTTP and cTTP, the aforementioned clinical features may vary for patients with cTTP. This review discusses cTTP-specific clinical features in comparison with those of iTTP.

Hence, we must investigate the appropriate treatment and management for better long-term outcomes, likely through enriched international cohort studies and clinical trials of novel therapies.

## 2. Diagnostic Flow for cTTP

Among patients with severe thrombocytopenia and hemolytic anemia of unknown cause, the reduction in ADAMTS13 levels to <10% of normal values confirms a diagnosis of TTP [23]. Two assays developed in Japan are commonly used to measure ADAMTS13 activity. The first is the chromogenic ADAMTS13 act-enzyme-linked immunosorbent assay (ELISA), in which the N10 monoclonal antibody directly detects the cleavage site of a synthetic 73-amino-acid peptide, VWF73 [24,25]. The second, the fluorescence resonance energy transfer (FRET)-VWF73 assay, detects increased fluorescence generated by the FRET-VWF73 substrate cleaved by ADAMTS13 in the plasma [26]. Recently, a fully automated assay, the HemosIL AcuStar ADAMTS13 activity assay, became available, which helps to more rapidly measure ADAMTS13 activity (33 min) [27]. However, measuring ADAMTS13 activity with the FRET-VWF73 assay requires careful attention as extremely high levels of serum bilirubin (>100 μmol/L, 5.85 mg/dL) can interfere with fluorescence evolution by acting as a quencher at an emission wavelength of 450 nm [28].

The detection of autoantibodies against ADAMTS13 using the Bethesda assay or anti-ADAMTS13 IgG ELISA is required to distinguish between iTTP and cTTP [29]. In cases of negative results, the assay must be repeated on samples drawn at different time points because patients at presentation of the acute TTP event sometimes do not present autoantibodies, probably due to immunocomplexes with ADAMTS13 and autoantibodies in circulation [30]. Measuring ADAMTS13 activity in the parents of affected individuals to confirm mild–moderate ADAMTS13 depletion, usually seen in individuals with heterozygous ADAMTS13 mutations [31], is also useful for cTTP diagnosis. 

A diagnosis of cTTP is confirmed through genetic analysis and identification of causative *ADAMTS13* mutations (homozygous or compound heterozygous). Polymerase chain reaction (PCR) direct sequencing, also known as Sanger’s method, is used to analyze the 29 exons [32]. Genetic confirmation is straightforward when the detected *ADAMTS13* mutations have been proven to be causative or pathogenic variants based on in vitro ADAMTS13 expression studies. In the Japanese registry data, 67 clinically diagnosed patients underwent genetic analysis, which identified 68 different mutations in 60 families as of May 2022; these mutations included missense mutations, nonsense mutations, insertions/deletions, structural variants, and aberrant splicing [31]. Comprehensive genomic quantitative PCR can compensate for the limitations of direct PCR sequencing [33]. Some complex cases may also show variants including copy number variants, deep-intronic splice site variants, repeat expansions, structural variants, or mobile element insertions. In addition, a recent in silico study showed that some synonymous single nucleotide variants (sSNVs) in *ADAMTS13* change mRNA folding energy/stability, disrupt mRNA splicing, disturb microRNA-binding sites, and alter synonymous codon or codon pair usage [34].

## 3. Current Treatments for Patients with cTTP

In most cases, cTTP diagnosis is not confirmed when patients experience their first episode of severe TTP because autoantibody detection via ELISA sometimes fails to distinguish between iTTP and cTTP [23]. FFP infusion is typically sufficient to achieve clinical remission [22]. However, these initial cases are generally treated with therapeutic plasma exchange (TPE), the standard therapy for iTTP. Based on previous experiences, acute TTP episodes in confirmed cTTP can be treated with several FFP infusions [22]. The exact amount of ADAMTS13 required to resolve acute TTP episodes has not been determined; however, sole FFP infusions can successfully adjust the unbalanced ADAMTS13-VWF axis in patients with cTTP compared to that in patients with iTTP with numerous neutralizing autoantibodies against ADAMTS13. The recent International Society on Thrombosis and Haemostasis (ISTH) TTP guidelines suggest daily plasma infusions for symptomatic patients until the symptoms resolve and platelet counts reach the normal range [35,36]. If the underlying trigger is treatable (e.g., bacterial or influenza virus infection), suitable medication should be administered in parallel to prevent further VWF secretion by the endothelial cells. However, the level of ADAMTS13 activity required to overcome acute TTP episodes remains unknown.

As maintenance therapy, the ISTH TTP guidelines recommend treating cTTP with FFP (10–15 mL/kg) every 1–3 weeks to prevent further acute episodes [35,36]. In the international hereditary TTP registry, 83 patients (70% of the total) received regular treatment, with a median interval of regular infusions of 14.0 days (range: 2–75 days) [19]. Another report on the annual incidence of TTP episodes reported a mean plasma volume dose of <15 mL/kg every 2 weeks in 79% (60 out of 76) of patients with available information [37]. In the UK registry, 67% of the patients received regular prophylactic therapy, 12% received on-demand therapy, and 21% had never received therapy since the initial diagnosis of cTTP. The interval between infusions was determined in a stepwise manner until once weekly. The single dosage of replacement therapy per body weight was not available [20]. In Japan, 240–480 mL of prophylactic FFP infusion every 2 weeks has been recommended over the past two decades based on our experience [38]. 

The decision to administer regular FFP is based on the opinion of the attending physician, with the aim of maintaining a sufficient platelet count. As mentioned above, large cohort studies revealed that not all patients receive prophylactic FFP infusions, and that up to 30% of patients require FFP infusions only if they develop thrombocytopenia due to triggers such as infection, trauma, or pregnancy (on-demand FFP infusions) [19,20,39]. For instance, some children do not receive FFP infusions because of the difficulty in finding suitable venous access. Patients also tend to reject prophylactic FFP infusion if they are not FFP-dependent (e.g., normal platelet count, no hemolytic anemia, and no recurrence of TTP episodes). 

**Figure 1:**
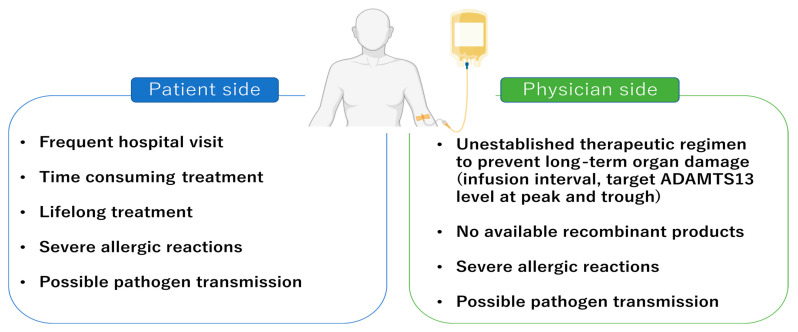
Current issues related to the use of prophylactic fresh frozen plasma infusion in patients with cTTP. Patients and physicians are concerned about several limitations of this treatment, which can decrease patients’ quality of life.

In patients requiring ADAMTS13 supplementation, prophylactic FFP infusion is often burdensome in multiple ways (Figure 1). First, FFP infusions are performed only in a hospital setting, frequently (once every 1–3 weeks). Patients outside urban areas may have to move closer to the city to continue receiving ADAMTS13 replacement therapy. Moreover, FFP administration takes several hours of infusion due to its high volume. Care is usually taken to avoid volume overload in patients with impaired cardiac or renal function. Second, FFP contains not only the ADAMTS13 protein but also other proteins that may cause allergic reactions and can potentially transmit pathogens to patients [39]. Allergic reactions can range from hives to life-threatening anaphylactic shock and are more apparent in patients receiving prophylactic FFP compared to those receiving on-demand FFP. Our registry data showed that 58% of patients receiving prophylactic FFP infusion required premedication against allergic reactions before each FFP infusion, including steroids (hydrocortisone, prednisolone, and betamethasone), antihistamine agents (d-chlorpheniramine maleate and hydroxyzine), and anti-allergic medicines (fexofenadine and epinastine) [39]. A UK group has demonstrated the efficacy of two plasma-derived factor VIII/VWF concentrates as a source of ADAMTS13 (Koate-DVI and BPL 8Y). Since these agents have smaller volume and fewer other plasma proteins than FFP, they can be helpful for small children or patients requiring desensitization because of intolerant hypersensitivity to FFP [40,41]. Notably, ISTH TTP guidelines do not recommend the use of FVIII concentrate for most patients with cTTP in remission because of lacking clear evidence about the variability of ADAMTS13 concentrations across various FVIII concentrate products with intermediate purity [35]. Solvent–detergent-inactivated and amotosalen-UVA pathogen-inactivated FFP can reduce severe allergic reactions [42,43,44]. However, these manipulated plasma products are not available in some countries.

In addition, a recent case report showed that a single dose of caplacizumab plus FFP infusion reduced the required FFP volume and hospital stay in refractory cTTP relapse [45]. Caplacizumab is a humanized nanobody that inhibits the interaction between VWF multimers and platelet glycoprotein 1b, and it has been approved for acute iTTP [46]. The treatment experience for cTTP is limited; however, it may benefit patients with refractory cTTP, sustained ischemic organ damage, and FFP intolerance due to allergic reactions. Moreover, caplacizumab has not yet been approved for cTTP as of March 2023.

## 4. Long-Term Organ Damage and Mortality in Patients with cTTP

The therapeutic regimen proposed in the ISTH guidelines mainly focuses on avoiding acute TTP episodes, including severe thrombocytopenia and ischemic organ damage. The most frequent acute TTP episodes during follow-up are of mild severity and are caused by acute infection [37]. However, the long-term organ damage in cTTP impacting the quality of life (QOL) of patients remains unclear (Figure 1). To clarify this risk among patients with cTTP, we conducted a questionnaire study in a Japanese cTTP cohort and analyzed the data from 55 eligible patients [39]. Forty-one patients received prophylactic FFP infusions (prophylactic FFP group), 14 of whom were included in the on-demand FFP group. In the prophylactic FFP group, the median dose of FFP infusion was 13.2 mL/kg/month, which was lower than that suggested in the ISTH guidelines (roughly 20–30 mL/kg/month). The trough levels of ADAMTS13 activity were available for 24 of 41 patients in the prophylactic FFP group, while 16 of these cases had levels below the detection limit (<1%). Laboratory findings immediately before FFP infusion revealed mild-to-moderate thrombocytopenia (median 138 × 10^9^/L). A total of sixteen patients developed organ damage. Chronic kidney disease (CKD) was observed in 13 patients; five had end-stage renal failure and required renal replacement therapy, four required hemodialysis, and one underwent renal transplantation from his father. Six patients developed cerebral infarction and one patient developed cardiac hypofunction, during follow-up. Another study showed that all 25 enrolled patients reported the presence of more than two neuropsychiatric symptoms including headaches, difficulty in concentration, and depression. Headaches with aura (presumed to be migraines), vision changes, forgetfulness, fatigue, neuropathy, dysarthria, loss of vision, seizures, transient weakness, falls, and dysphagia were also reported [47]. Notably, 17 of the 25 patients developed strokes as they aged, and 11 had stroke-related sequelae. Stroke can occur in two different ways: during acute TTP episodes or from a gradual occlusion of cerebral vessels by latent microthrombi; however, which mechanism occurs more commonly in cTTP is not known. In another literature review, 202 patients identified between 2001 and 2020 were analyzed for their morbidities. Among those over 40 years of age, 20 (51%) had a major comorbidity, and 11 (28%) patients experienced a recurrence of a major morbidity after starting prophylactic FFP [48]. 

In contrast, in our previous study, none of the 14 patients with asymptomatic cTTP, who maintained platelet count within the normal range without regular FFP infusions, developed the kind of organ damage described above [39]. Indeed, the median serum creatinine level in this group was significantly lower than that among FFP-dependent patients (0.58 mg/mL vs. 0.71 mg/mL, *p* = 0.009). The trough levels of ADAMTS13 activity were available for only seven patients, and three cases were below the detection limit (<1%). Thus, these asymptomatic patients did not show relatively higher levels of ADAMTS13 activity, although previous studies showed that residual ADAMTS13 activity prevented more acute TTP episodes. These findings are summarized in Table 1.

As of May 2022, 10 of the 68 patients in the Japanese cTTP registry had died during follow-up. A young female patient who had received prophylactic FFP infusions after her first TTP episode during pregnancy committed suicide. Except for this case, the 10-year overall survival rate after the clinical diagnosis was 91.1% [31]. The causes of death are summarized in Table 2. The median patient age at the time of death was 44 years (IQR: 41–52 years). Two patients died due to thrombosis-related events. Five other patients died suddenly, suggesting abrupt cardiopulmonary dysfunction such as fatal arrhythmia or heart failure due to myocardial infarction. Three of the sudden death cases (60%) were in renal replacement. Previous studies on non-cTTP patients undergoing dialysis revealed a 2.2-fold higher mortality in patients with elevated VWF antigen levels compared to patients without elevated antigen levels [49]. Up to one-quarter of non-cTTP patients undergoing hemodialysis died of sudden cardiac death, suggesting that hemodialysis is associated with ventricular arrhythmia and dynamic electrocardiographic changes [50,51]. In patients with cTTP, more chronic glomerular sclerotic changes with C4d deposits were identified in the histopathological findings of renal biopsies with progressive renal impairment compared to controls, suggesting that C4d immunostaining provides evidence for complement-mediated glomerular damage in patients with cTTP [52]. The follow-up data from the international hTTP registry also showed that 4 of 87 patients died during prospective follow-up due to large cerebral infarction, heart failure, lethal arrhythmia with asystole during sepsis, and death from an unknown cause, respectively [37]. 

Considering this limited information, stroke and progressive renal failure during long-term follow-up substantially affect patients’ QOL. Although the amount and frequency of ADAMTS13 supplementation needed to prevent long-term organ damage in patients with FFP-dependent cTTP is not well known, further investigations based on the clinical use of recombinant ADAMTS13 products and large-scale cTTP cohorts are needed to address this unmet medical need. In addition, we must determine whether asymptomatic patients with cTTP can be followed up without ADAMTS13 replenishment.

## 5. Determinants of FFP-Dependent or Asymptomatic Phenotypes in cTTP

Intriguingly, some patients with cTTP require regular FFP infusions to avoid severe thrombocytopenia and ischemic organ damage due to TTP episodes, whereas others seem to be free from TTP episodes unless a triggering factor is present. Schulman et al. reported a case of a 9-year-old patient who could not maintain platelet counts within the normal range without FFP [14], and Upshaw et al. described a 29-year-old patient who developed thrombocytopenia and petechial rash only when she had an infection [15]. These patients were classified as having FFP-dependent cTTP and asymptomatic cTTP, respectively. As acute TTP episodes triggered by infection [37] or pregnancy [53,54] have received increasing attention, there are few observations regarding FFP dependency after recovery from acute TTP episodes.

To clarify the determinants of FFP-dependent or asymptomatic phenotypes in cTTP, we hypothesized that the clinical presentation is derived from different genotypes (*ADAMTS13* mutations). As mentioned above, cTTP causative mutations are distributed throughout the ADAMTS13 sequence, predominantly R193W in the metalloprotease domain and C908Y in the TSP5 domain [31]. However, the presence of these mutations has not been shown as evidence of FFP dependency. c.4143_4144dupA (p.E1382Rfs*6) and p.R1060W are commonly observed in patients in Western countries [19,20], and a recent report from the international hTTP registry indicated that 12 of 87 patients who were compound heterozygous carriers of p.R1060W mutations had a residual ADAMTS13 activity of 1–9% and had a low incidence rate of acute episodes [37].

We sometimes encounter siblings with the same ADAMTS13 mutations presenting with different clinical pictures, suggesting that the cTTP severity might be determined not only by the type of ADAMTS13 mutation but also by other underlying factors [37,39].

## 6. Emerging Novel Therapies Will Improve the QOL and Long-Term Outcomes of Patients with cTTP

Current FFP dosages vary widely depending on patient characteristics and physician preferences, suggesting that it is difficult to establish an equal standard of care for all patients. Recombinant ADAMTS13 concentrate (r-ADAMTS13; codename: TAK-755) was developed in the early 2010s. The efficacy of r-ADAMTS13 was proven in cTTP mice, in which acute TTP was induced using a VWF concentrate. r-ADAMTS13 prevented severe thrombocytopenia and microthrombi in systemic tissues [55]. In the phase I first-in-human clinical trial, the pharmacokinetic (PK) profiling of r-ADAMTS13 was similar to that of FFP in a dose-dependent manner [56]. Its safety, immunogenicity, and tolerability were also demonstrated in the participants. Notably, patients were free from the physical burden and intolerant allergic reactions compared to regular FFP treatments [57]. Similar to hemophilia therapy, r-ADAMTS13 home infusion will become more common in patients with cTTP, regardless of generation. A phase III international multicenter clinical trial is currently underway to identify the side effects of long-term treatment with r-ADAMTS13 (https://www.clinicaltrials.gov/ct2/show/NCT04683003, accessed on 1 February 2023). Very recently, two important papers from a phase III trial described the efficacy and safety of r-ADAMTS13 in severe neonatal and pregnancy cases [58,59]. r-ADAMTS13 will soon be approved for clinical use and enable more patients with cTTP to easily maintain much higher ADAMTS13 activity at peak and trough levels. Meanwhile, additional clinical information on prophylactic r-ADAMTS13 will supply more robust evidence of long-term preventable effects against recurrent acute TTP episodes and progressive organ damage. 

ADAMTS13 was also shown to down-regulate platelet adhesion to the exposed subendothelium and thrombus formation in injured arterioles [60] and reduce ischemic brain injury in experimental stroke [61,62]. Moreover, recombinant ADAMTS13 reduced oxidative stress by cleaving VWF in ischemia/reperfusion-induced acute kidney injury [63]. Even in healthy individuals, a slight decrease in ADAMTS13 activity (<70%) is a risk for stroke [64]. The anti-thrombotic effects of ADAMTS13 have also been described in myocardial infarction [65], chronic thrombotic pulmonary hypertension [66], and inflammatory bowel disease [67]. Hence, prophylactic r-ADAMTS13 infusion can benefit asymptomatic patients with cTTP, although data from our Japanese cohort showed no long-term organ damage in this group. 

Regarding the progress of treatments for patients with hemophilia A and B, longer-acting recombinant factor concentrates have become widely available, and therapeutic intervals have been extended to maintain suitable factor levels since mid-2010. In November 2022, the U.S. Food and Drug Administration (FDA) approved Hemgenix (etranacogene dezaparvovec), an adeno-associated virus (AAV) vector-based gene therapy for the treatment of adults with HB who currently use Factor IX prophylaxis therapy or have a history of life-threatening hemorrhage, or have repeated, severe spontaneous bleeding events [68]. Although gene therapy has been thought of as a costly treatment, a recent study reported that a single use of gene therapy could compensate for lifelong consecutive factor prophylaxis [69]. Hence, gene therapy could also overcome the limitation of short-term ADAMTS13 replacement because the ADAMTS13 transgene would allow the long-term expression of ADAMTS13 and free cTTP patients from lifelong replacement therapy [70]. Some research groups have already shown promising long-term ADAMTS13 or MDTCS expression in *ADAMTS13* knock-out mice via different applications, including hematopoietic progenitor-cell transgene, in utero gene transfer of the lentiviral vector, adenoviral vector-mediated transgene, AAV-mediated transgene, and sleeping-beauty transposon-mediated gene transfer [71,72,73,74,75]. Therefore, gene therapy for cTTP may be a reasonable therapeutic option once its long-term efficiency and safety have been established.

## 7. Conclusions

This review discussed in detail the clinical manifestations of the very rare congenital TTP condition, the challenges with current plasma therapy, and the long-term prognosis based on the latest reports. Physicians treating patients with FFP often use platelet counts as a basis because the link between the peak/trough levels of ADAMTS13 activity and long-term organ damage has not been well investigated. Collecting data from cTTP registries could offer greater insights into morbidity and mortality during long-term follow-up. The emerging novel r-ADAMTS13 product has the potential to keep ADAMTS13 activity higher than conventional FFP infusions and improve the QOL. Further investigations are necessary to determine if r-ADAMTS13 could improve long-term outcomes in patients with cTTP.

## Figures and Tables

**Table 1:** Long-term consequences reported in follow-up studies on congenital thrombotic thrombocytopenic purpura (cTTP).

Summary	Reference
The prophylactic FFP group received a lower FFP dose than suggested in the ISTH guidelines (13.2 mL/kg/month vs. 20–30 mL/kg/month). These patients showed mild thrombocytopenia immediately before FFP infusions (median 138 × 10^9^/L). Chronic kidney disease was the most prevalent organ damage among these patients (32%), followed by cerebral infarction (15%) and cardiac hypofunction (2%) during follow-up.	Japanese registry [39]
Sixty-eight percent of patients developed strokes as they aged and 44% had stroke-related sequelae.	Oklahoma registry [47]
Among patients over 40 years of age, 51% had a major comorbidity, and 28% of patients experienced a major morbidity recurrence after initiating prophylactic FFP.	Literature review [48]
The FFP on-demand group maintained platelet counts within a normal range without regular treatment and did not develop long-term organ damage during follow-up.	Japanese registry [39]

**Table 2:** Causes of death among patients in the Japanese cohort with congenital thrombotic thrombocytopenic purpura (cTTP).

Code	Age at Death (Years)	Sex	Follow-Up (Years)	Cause of Death	Renal Impairment	Complications/ Backgrounds	Prophylactic FFP Infusion
C3	38	M	30	Unknown *	ESRD (HD)		Yes
H3	52	M	1	Uremia	ESRD (HD)	GIH	No
R5	37	F	14	Suicide			Yes
X5	44	F	4	Unknown *		SLE	No
2G2	79	M	3	Cerebral infarction			Yes
2N4	23	F	0	Unknown *		Pregnancy	No
2O	41	M	2	Unknown *	ESRD (HD)		No
2P4	44	F	17	Status epilepticus, NOMI		Paralysis after stroke	Yes
2R	48	M	14	Unknown *	ESRD (HD)		Yes
2T	66	M	1	Sepsis	ESRD (HD)		Yes

* Sudden death, suggesting sudden cardiac death. Abbreviations: NOMI, non-occlusive mesenteric ischemia; ESRD, end-stage renal disease; HD, hemodialysis; GIH, gastrointestinal hemorrhage; SLE, systemic lupus erythematosus; FFP, fresh frozen plasma.

## Data Availability

Not applicable.
